# Addendum to consensus opinion from the International Deep Endometriosis Analysis (IDEA) group: sonographic evaluation of superficial endometriosis

**DOI:** 10.1002/uog.29288

**Published:** 2025-07-09

**Authors:** S. Guerriero, G. Condous, M. Rolla, M. Pedrassani, M. Leonardi, G. Hudelist, S. Ferrero, J. L. Alcazar, S. Ajossa, C. Bafort, D. Van Schoubroeck, T. Bourne, T. Van den Bosch, S. S. Singh, M. S. Abrao, A. Di Giovanni, C. Tomassetti, D. Timmerman

**Affiliations:** ^1^ Centro Integrato di Procreazione Medicalmente Assistita (PMA) e Diagnostica Ostetrico‐Ginecologica, Azienda Ospedaliero Universitaria di Cagliari ‐ Policlinico Duilio Casula Monserrato Italy; ^2^ Department of Surgical Sciences University of Cagliari Cagliari Italy; ^3^ Acute Gynaecology, Early Pregnancy and Advanced Endoscopy Surgery Unit, Sydney Medical School Nepean, Nepean Hospital University of Sydney Penrith New South Wales Australia; ^4^ Department of Gynecology and Obstetrics of Parma University of Parma Parma Italy; ^5^ CLINUS Ultrasound Clinic Florianópolis Brazil; ^6^ Department of Obstetrics and Gynecology Hospital Maternidade Carmela Dutra Florianópolis Brazil; ^7^ Department of Obstetrics and Gynecology McMaster University Hamilton ON Canada; ^8^ Department of Gynecology Center for Endometriosis, Hospital St John of God Vienna Austria; ^9^ Rudolfinerhaus Private Clinic and Campus Vienna Austria; ^10^ Department of Neurosciences, Rehabilitation, Ophthalmology, Genetics, Maternal and Child Health (DiNOGMI) University of Genoa Genoa Italy; ^11^ Academic Unit of Obstetrics and Gynecology, IRCCS Ospedale Policlinico San Martino Genoa Italy; ^12^ Department of Obstetrics and Gynecology School of Medicine, Universidad de Navarra Pamplona Spain; ^13^ Department of Obstetrics and Gynaecology, Leuven University Fertility Centre University Hospitals Leuven Leuven Belgium; ^14^ Department of Development and Regeneration KU Leuven Leuven Belgium; ^15^ Queen Charlotte's and Chelsea Hospital Imperial College London London UK; ^16^ Department of Obstetrics and Gynecology The Ottawa Hospital Ottawa Canada; ^17^ Gynecologic Division, BP‐A Beneficencia Portuguesa de São Paulo São Paulo Brazil; ^18^ Disciplina de Ginecologia, Departamento de Obstetricia e Ginecologia Hospital das Clinicas da Faculdade de Medicina da Universidade de São Paulo São Paulo Brazil; ^19^ Endoscopica Malzoni, Malzoni Research Hospital Avellino Italy

## Abstract

Traditionally, laparoscopy was considered to be the gold standard for the examination of endometriotic lesions, because it allows their direct visualization. Several international and national guidelines have now shifted their focus to non‐invasive imaging‐based diagnosis of deep endometriosis in preference to surgery, while laparoscopy is used to diagnose superficial endometriosis in patients with painful symptoms and negative ultrasound and/or magnetic resonance imaging findings for ovarian or deep endometriosis. In recent years, however, the role of gynecological ultrasound in the diagnosis of superficial endometriosis has been studied more extensively. The purpose of this addendum to the International Deep Endometriosis Analysis consensus opinion is to describe a standardized ultrasound protocol for the diagnosis of superficial endometriosis and to highlight the sonographic characteristics of these lesions. © 2025 The Author(s). *Ultrasound in Obstetrics & Gynecology* published by John Wiley & Sons Ltd on behalf of International Society of Ultrasound in Obstetrics and Gynecology.

## INTRODUCTION

Endometriosis is a systemic inflammatory condition associated with the presence of displaced endometrium‐like tissue outside the uterus, and affects 10–15% of women and individuals assigned female at birth^1^. Although endometriosis may be asymptomatic in some women, it can alter the quality of life of others, as it can be associated with chronic pelvic pain and subfertility or infertility in 35–50% of cases.

The diagnosis of endometriosis can be challenging. Prior to the current era of increased awareness of endometriosis among patients and physicians, diagnosis was often delayed^2^. Laparoscopy was considered to be the gold standard, as it allows direct visualization of endometriotic lesions. Recently, several national and international guidelines^3^, ^4^, ^5^, ^6^ have shifted the focus to non‐invasive imaging‐based diagnosis, which is preferred over surgery. Early and accurate diagnosis ensures early treatment, which may prevent progression of the disease and improve patient outcomes^7^. The International Deep Endometriosis Analysis (IDEA) consensus opinion standardized the ultrasound examination of patients with endometriosis^8^.

There are three phenotypes of endometriosis, which can be isolated or synchronous: superficial, deep and ovarian^9^. Using the standardized ultrasound protocol of the IDEA consensus^8^, excellent accuracy for the diagnosis of deep and ovarian endometriosis is possible, while the diagnostic accuracy for superficial endometriosis is limited. Superficial endometriosis, also known as peritoneal endometriosis, is defined as the presence of endometrium‐like tissue lesions involving the peritoneal surface^10^. The lesions can have different appearance and color (e.g. clear, black, flat, cystic)^1^. The prevalence of superficial endometriosis is not known, but it has been found in 80% of women with endometriosis^11^ and in 40% of asymptomatic patients (data obtained in a group of fertile, asymptomatic women undergoing laparoscopy for sterilization)^12^. It may be associated with pelvic pain and infertility, with adjusted prevalence ratios of 1.83 for primary infertility, 1.43 for severe dysmenorrhea and 1.50 for deep dyspareunia^13^.

While the 2022 guidelines of the European Society of Human Reproduction and Embryology (ESHRE) emphasized the role of ultrasound and magnetic resonance imaging (MRI) in the diagnosis of endometriosis, they acknowledged the limitations in detecting the presence of superficial endometriotic lesions and recommended that laparoscopy should be used to diagnose superficial endometriosis in patients with painful symptoms and negative ultrasound findings for ovarian or deep endometriosis^3^. However, the role of gynecological ultrasound in the diagnosis of superficial endometriosis has been studied extensively in more recent years.

The purpose of this consensus opinion is to describe a standardized ultrasound protocol for the diagnosis of superficial endometriosis.

## METHODS

This work is based on the opinion of a multidisciplinary panel of 18 clinicians, gynecological sonologists and advanced laparoscopic surgeons (the IDEA group). All had expertise in the diagnosis and management of endometriosis and significant peer‐reviewed publications in the field. An initial statement was prepared (S.G., M.R., J.L.A.) as a first draft in May 2024 and sent to all panel members. They were given the opportunity to comment within a specified timeframe and were required to respond. Taking into account all comments, a revised draft was then produced. In case of conflicting opinions, a consensus was proposed after discussion among at least three members of the expert panel, and this process was repeated until consensus was reached among all the experts. This consensus also presents ultrasound images and schematic drawings to illustrate the text. After four rounds of revision, the manuscript was considered ready for publication.

In addition to terms and definitions to describe the sonographic features of superficial endometriosis, this consensus provides recommendations on how and in whom to perform an ultrasound examination in patients with suspected superficial endometriosis.

## ANATOMOPATHOLOGICAL FINDINGS

Early lesions of superficial endometriosis are transparent on laparoscopy, prior to the development of neoangiogenesis. Subsequently, owing to peritoneal inflammatory mechanisms, there is secretion of estrogen and inflammatory mediators that stimulate mitosis and neoangiogenetic processes. At this stage, peritoneal endometriotic lesions appear red. As the process of hemoglobin deoxygenation to methemoglobin progresses, the peritoneal lesion changes from red to black in color. Black lesions then become white owing to the accumulation of biliverdin, bilirubin and fibrous tissue. The lesions then become smaller as the stroma is replaced by fibrous tissue^14^. Old lesions eventually disappear, leaving room for new ones to form, along with newly developed fibrotic tissue. In summary, superficial endometriosis can be visualized on laparoscopy as powder‐burn lesions, red flame‐like lesions, peritoneal pockets or clear vesicles^15^ (Figure 1a).

**Figure 1:**
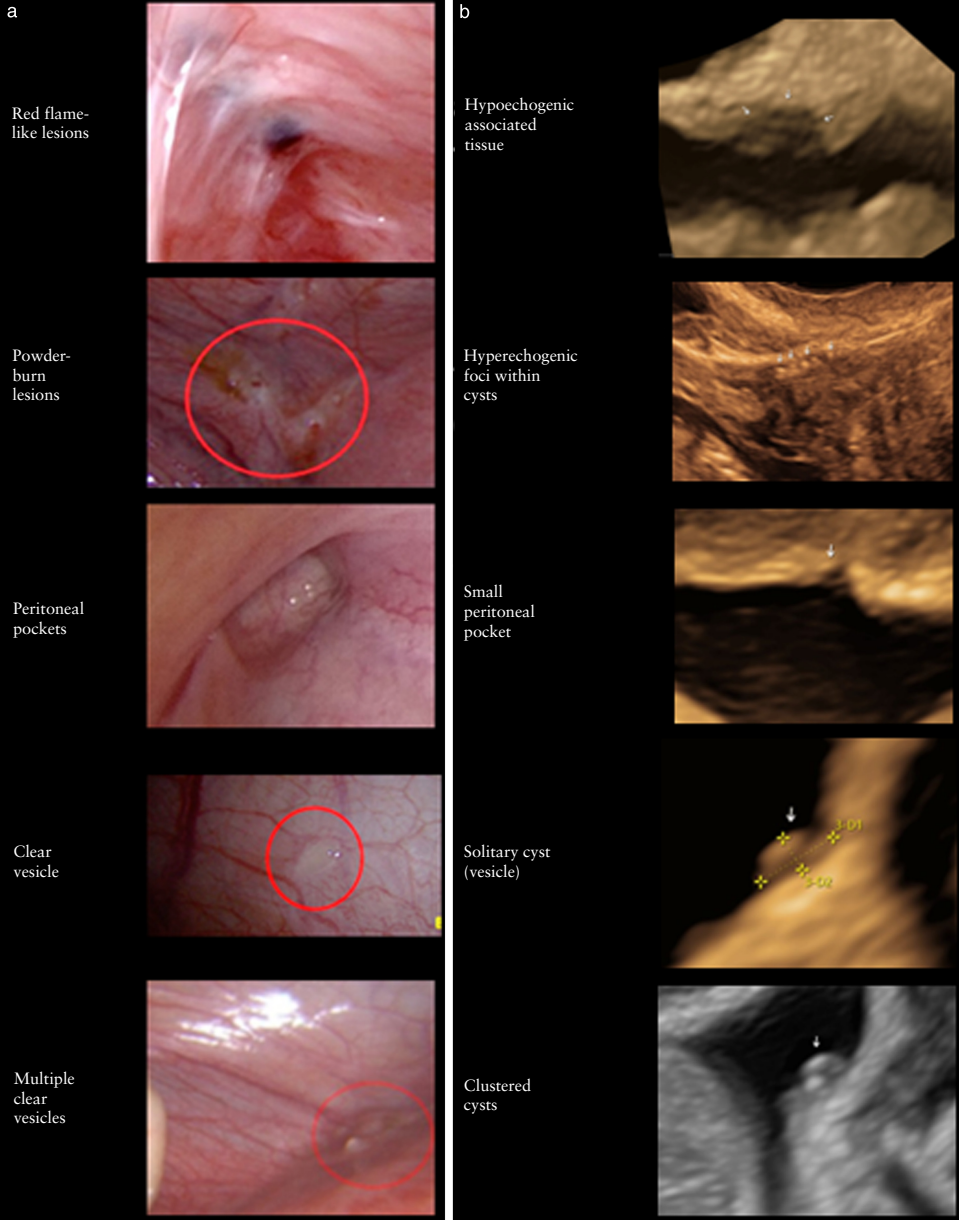
(a) Appearance of superficial endometriosis at laparoscopy (based on Van Langendonckt *et al*.^15^) and (b) corresponding ultrasonographic findings (based on Pedrassani *et al*.^7^, Leonardi *et al*.^16^ and Bailey *et al*.^25^).

## SONOGRAPHIC ASSESSMENT OF SUPERFICIAL ENDOMETRIOSIS

Although superficial endometriosis has traditionally been considered difficult to detect by ultrasound, advances in technology and techniques have improved the accuracy and reliability of this method for diagnosing superficial endometriosis.

### Who should be scanned for superficial endometriosis

A critical question is which patients would benefit from ultrasound evaluation for superficial endometriosis. From a clinical point of view, it would not make sense to assess the presence of superficial endometriosis by either ultrasound or MRI in women who have already been diagnosed with deep endometriosis or ovarian endometriosis. In these women, the likelihood of having superficial endometriosis is high^13^. However, approximately 29% of women with endometriosis will have only superficial endometriosis^13^. We recommend this specific evaluation in patients with symptoms, such as pain, that are suggestive of endometriosis and/or infertility, in the absence of ultrasound features of deep or ovarian endometriosis.

### How to scan for superficial endometriosis

A transvaginal ultrasound examination of patients with endometriosis should be performed in a standardized manner, as described by the IDEA group^8^. Assessment of the anterior, central, posterior and lateral compartments should be performed consecutively and continuously, focusing on the surface of the peritoneum and the serosa of the pelvic organs. It is particularly important to evaluate the peritoneum of the pouch of Douglas (POD), as this is the most common site of superficial endometriosis^16^, and because it is the easiest location to evaluate using ultrasound. For this task, the presence of fluid in the POD facilitates ultrasound evaluation. In many women, at least a small amount of fluid is present in the POD throughout the natural menstrual cycle, although this is most common during the periovulatory and menstrual periods. However, in women undergoing hormonal treatment, the presence of fluid may be less likely and the amount of fluid smaller. It should be borne in mind that, in the postmenstrual period, the presence of clots and fibrin may be a source of false‐positive results. In the absence of fluid in the POD, sonoPODography may be considered^16^. This is an ultrasound procedure associated with the instillation of saline into the POD through an intrauterine balloon catheter as in sonohysterography, but with instillation of additional fluid, and it enables direct observation of superficial endometriosis while avoiding the use of hyperechogenic contrast medium. The transvaginal probe should be positioned within the posterior vaginal fornix in the midsagittal plane, regardless of whether the uterus is anteverted or retroverted. In an anteverted uterus, the cervix is displaced anteriorly and is not in view when evaluating the posterior compartment structures.

Without compressing the POD with the transducer, the exam should begin with its evaluation, through the peritoneum. Even a small pocket of fluid can be visualized. The fluid can be brought to the posterior region of the abdomen by push–pull maneuvers with the transducer. Compression of the patient's abdomen using the other hand often helps to move fluid to the lateral compartments and to move the ovary in order to evaluate the ovarian fossae. The ovarian follicles can help as an acoustic window to visualize the peritoneum in the fossae. If fluid is present in the anterior vesicouterine pouch, superficial endometriosis can also be visualized there, with the probe placed in the anterior vaginal fornix and using a gentle retraction technique to ensure that the fluid settles and is not displaced from this space. Scanning must be slow, in the sagittal, axial and transverse planes. Scanning tangentially to the peritoneum, especially in the presence of fluid, helps to visualize superficial lesions in all compartments.

It is unclear whether intestinal preparation prior to examination helps in the evaluation of superficial endometriosis^17^, ^18^. Therefore, until further data are available, it is preferable to avoid this additional procedure, to facilitate the tolerability and accessibility of the endometriosis scan.

#### Technical issues

Use of a high‐end ultrasound machine is recommended, as high proximal resolution is required to visualize structures smaller than 5 mm. It is recommended that the highest possible frequency be used, with second harmonic or inverted pulse harmonic imaging. The use of spatial compounding techniques of acquiring and combining ultrasound information in real time is also recommended to reduce speckle, and to zoom in on the lesion to obtain maximum image detail (the lateral scale on the monitor should be between 2 and 4 cm).

#### Indirect signs of superficial endometriosis (‘soft markers’)

In 2018, Robinson *et al*.^19^ attempted to evaluate the use of three markers on transvaginal ultrasound (thickening (‘white‐line’ sign), tenderness and small hypoechogenic nodules) to predict superficial endometriosis near the uterosacral ligaments in women with symptoms of endometriosis, but concluded that their use was not clinically useful as a screening test. In 2019, Reid *et al*.^20^ conducted a multicenter prospective observational study to investigate whether two ultrasound soft markers (ovarian mobility and site‐specific tenderness) were associated with the type of endometriosis (superficial, deep or ovarian) and location of the disease, in 189 patients undergoing surgery. They demonstrated that the isolated presence of superficial endometriosis in the pelvis or uterosacral ligaments correlated with the ultrasound finding of ovarian immobility with 71% diagnostic accuracy, 16% sensitivity and 87% specificity for the left side and 82% diagnostic accuracy, 7% sensitivity and 94% specificity for the right side. There was a statistically significant association between left adnexal site‐specific tenderness and superficial endometriosis of the left pelvic sidewall (*P* = 0.027) in the absence of endometrioma, deep endometriosis or POD obliteration (*n* = 112)^20^. In the same year, Chowdary *et al*.^21^ analyzed retrospectively the ultrasound findings of 53 patients who underwent surgery for pain and suspected endometriosis. In the patients with superficial endometriosis, positive ultrasound findings included thickened uterosacral ligament (> 3 mm) and thickened pericolic fat. However, this study has been criticized because the indirect sonographic sign of thickened pericolic fat was not well explained, rendering it difficult to reproduce^22^. In 2022, Rao *et al*.^23^ evaluated retrospectively the preoperative ultrasound findings of 77 patients who underwent surgery for suspected endometriosis and found that ovarian fixation played a diagnostic role in predicting moderate and severe stages of the disease. Subsequently, in 2023, Deslandes *et al*.^24^ conducted a prospective longitudinal cohort study in which they analyzed the diagnostic accuracy of ultrasound in the preoperative assessment of the type and location of endometriosis in 53 patients. Contrary to earlier findings by Reid *et al*.^20^, they found that the use of ultrasound soft markers (ovarian mobility) had limited diagnostic performance in detecting superficial endometriosis. In conclusion, based on the literature, the usefulness of indirect sonographic signs in raising suspicion of superficial endometriosis is limited.

#### Direct signs of superficial endometriosis

In 2020, Leonardi *et al*.^16^, in a prospective diagnostic accuracy study of sonoPODography, described sonographic superficial endometriotic lesions (confirmed surgically and histologically) as hyperechogenic projections, hypoechogenic areas, filamentous adhesions, cystic areas or peritoneal pockets. Pedrassani *et al*.^7^, in a prospective study of 52 patients, evaluated preoperatively, using transvaginal ultrasound, superficial endometriotic lesions that were confirmed laparoscopically. They assessed the peritoneal surfaces of the anterior, central and lateral pelvic compartments and described in detail the sonographic appearance of the lesions. The authors suggested that superficial endometriotic lesions may be suspected on ultrasound in the presence of hypoechogenic tissue surrounding a small cystic area (the tissue neither invaginated nor protruding from the peritoneal surface), a convex lesion protruding from the peritoneal surface into the peritoneal cavity or a concave lesion with a defect in the peritoneum, with associated hyperechogenic foci and/or velamentous (filmy) adhesions^7^.

Based on the descriptions by Pedrassani *et al*.^7^ and Leonardi *et al*.^16^, it was agreed that superficial endometriotic lesions have the following ultrasound characteristics (Figure 1b): (1) hypoechogenic tissue, associated with the presence of stromal reaction/fibrosis; (2) hyperechogenic foci within cysts, associated with the presence of hemosiderin deposit; (3) small or large peritoneal pocket, associated with vesicle(s); (4) solitary cyst (vesicle), associated with the presence of clear or red vesicles at laparoscopy; or (5) clustered cysts associated with the presence of multiple clear or red vesicles at laparoscopy. These lesions may appear as a single lesion, multiple lesions (apparently normal peritoneum may be seen between the lesions) or lesions organized in clusters (linear or honeycomb appearance). The presence of velamentous adhesions may be associated with any of these five types of lesion, but is not specific for the presence of superficial endometriosis (Figure 2).

**Figure 2:**
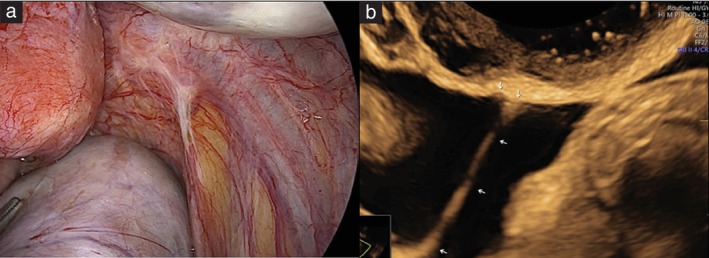
Velamentous (filmy) adhesions at laparoscopy (a) and on ultrasound (b, arrows).

Once a superficial endometriotic lesion has been identified, it should be measured and, if several lesions are present, they should be counted if possible. Usually, superficial lesions are a few mm in length (ranging from 1 to 5 mm) and up to 5 mm in depth. The location (usually the POD) should also be included in their description. Figure 3 summarizes the different ultrasonographic findings associated with the presence of superficial endometriosis. Examples of the sonographic appearance of superficial endometriosis are shown in Figure 4.

**Figure 3:**
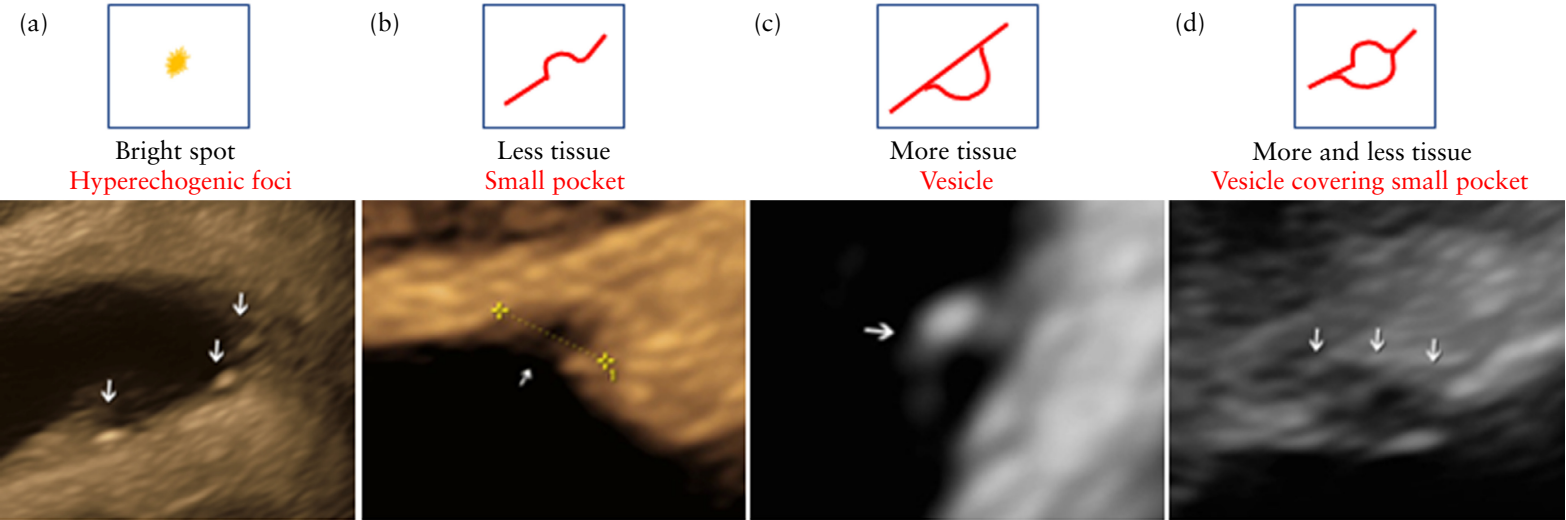
Summary of ultrasonographic findings associated with the presence of superficial endometriosis. Findings may appear in isolation (a,b,c) or in combination (d), and different lesions may show different associated findings within the same patient.

**Figure 4:**
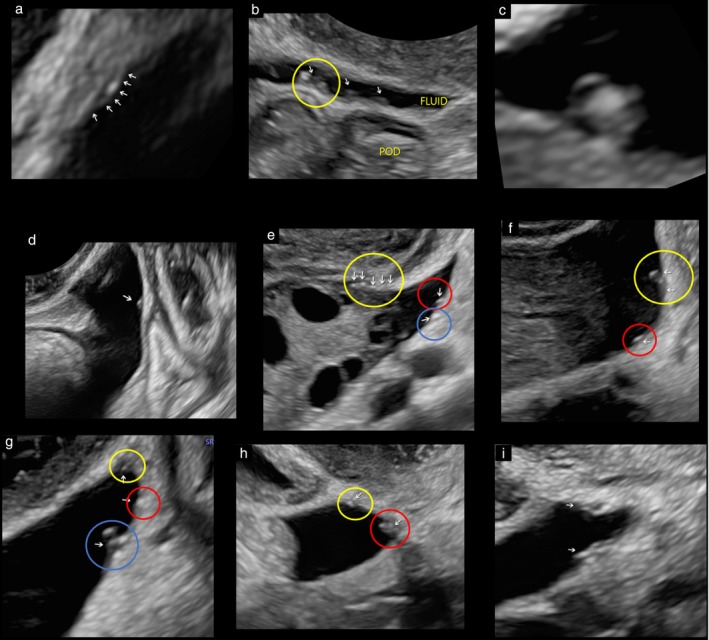
Examples of the ultrasound appearance of superficial endometriosis: (a) clustered linear cysts (arrows) with a hyperechogenic focus; (b) clustered tiny cyst (yellow circle and arrow) and two solitary cysts (arrows); (c) clustered tiny cysts with a honeycomb appearance; (d) solitary cyst (vesicle) (arrow); (e) clustered tiny cysts with a linear appearance (yellow circle and arrows), solitary tiny cyst (red circle and arrow) and solitary tiny cyst in a peritoneal small pocket (blue circle and arrow); (f) clustered tiny cysts with a honeycomb appearance (yellow circle and arrows) and solitary tiny cyst (red circle and arrow); (g) clustered tiny cysts with a hypoechogenic associated lesion (yellow circle and arrow), solitary cyst with a hyperechogenic focus in a small peritoneal pocket (red circle and arrow) and clustered cysts with a honeycomb appearance (blue circle and arrow); (h) solitary tiny cyst with hypoechogenic associated lesion and hyperechogenic focus (yellow circle and arrow) and clustered tiny cysts (red circle and arrow); (i) clustered cysts with a honeycomb appearance (arrows). POD, pouch of Douglas.

### Diagnostic performance of ultrasound in detection of superficial endometriosis

Leonardi *et al*.^16^ reported that sonoPODography detected lesions of superficial endometriosis confirmed at laparoscopy with a diagnostic accuracy of 69%, a sensitivity of 65% and a specificity of 100%. They also observed that, in the absence of deep, ovarian or obliterating endometriosis, ultrasound performance reached an accuracy of 80%, with a sensitivity of 78% and a specificity of 100%. In 2024, Bailey *et al*.^25^ evaluated the diagnostic role of ultrasound in the detection of superficial endometriosis in patients undergoing surgical treatment in a retrospective study of 100 patients. Specifically, their analysis of the location of superficial endometriosis was based on the presence of hypoechogenic lesions in the peritoneum of the POD. They reported that ultrasound showed a specificity of 96%, a sensitivity of 50% and a diagnostic accuracy of 87% in detecting superficial endometriotic lesions at the level of the peritoneum of the POD.

### Differential diagnosis

When diagnosing superficial endometriosis by ultrasound, it is important to keep in mind that similar imaging findings may be caused by other entities, such as postoperative adhesions, sequelae of pelvic inflammatory disease, menstrual clots or even peritoneal carcinomatosis. The possibility of imaging artifacts should also be considered. The clinical history should be contextualized and, in the presence of any potentially inflammatory source in the pelvis, it is advisable to err on the side of caution and not be conclusive in diagnosing superficial endometriosis, instead considering this as part of the differential diagnosis.

## DISCUSSION

Historically, the diagnosis of superficial endometriosis, a possible cause of pelvic pain and infertility^4^, has been possible only through laparoscopy. In the present addendum to the IDEA consensus opinion^8^, we propose how to describe superficial endometriosis using transvaginal ultrasound, based on recently published studies. Superficial endometriosis appears as hypoechogenic tissue with cystic areas, convex or concave lesions and hyperechogenic foci that may be associated with velamentous adhesions. They may be single, multiple or clustered lesions. We suggest that this evaluation be performed in patients with symptoms suggestive of endometriosis and/or infertility, in the absence of ultrasound features of deep or ovarian endometriosis. The presence of fluid in the pelvis enhances visualization of these very small lesions, which are found mainly in the POD and the vesicouterine space.

This addendum to the IDEA consensus opinion recommends standardization of the ultrasound examination and detailed description of superficial endometriosis, which could be utilized in future large, prospective studies of the disease. Unfortunately, the lack of such studies prevents evaluation of the true correlation between ultrasound findings and the clinical presentation of superficial endometriosis, increasing the risk of misdiagnosis, including both false positives and false negatives. In addition, the lack of information regarding the differential diagnosis between superficial endometriosis and other diseases, such as pelvic infection, tuberculosis and postoperative lesions, or imaging artifacts, may affect the clinical recommendations when findings of superficial endometriosis are observed on ultrasound examination. Future studies should investigate the accuracy of individual ultrasound features in the diagnosis of superficial endometriosis. From a technical point of view, it is important to consider that, as we ascend in the pelvis, it is more difficult to visualize the peritoneal surfaces, as there is less fluid, and more structures lie against the peritoneal surfaces.

In conclusion, this addendum to the IDEA consensus opinion provides recommendations on how and in whom to perform ultrasound examination for the diagnosis of superficial endometriosis, and highlights the sonographic characteristics of these lesions. However, in the absence of large, prospective studies, care must be taken to avoid the risk of overdiagnosis of this disease.

## Data Availability

Data sharing not applicable to this article as no datasets were generated or analysed during the current study.
